# Disparities in the Place of Death for Patients With Malignant Neoplasms of the Thyroid Gland

**DOI:** 10.7759/cureus.55506

**Published:** 2024-03-04

**Authors:** Fnu Anupiya, Preyansh K Doshi, Neera Vora, Bhavya Parekh, Suppraja Soundarrajan, Alousious Kasagga, Fnu Iffath Muneer Ahmed

**Affiliations:** 1 Internal Medicine, Kalinga Institute of Medical Sciences, Bhubaneswar, IND; 2 Internal Medicine, Gujarat Cancer Society Medical College, Hospital & Research Centre, Ahmedabad, IND; 3 Internal Medicine, Government Medical College Bhavnagar, Bhavnagar, IND; 4 Internal Medicine, Government Medical College, Omandurar Government Estate, Chennai, IND; 5 Pathology, California Institute of Behavioral Neurosciences & Psychology, Fairfield, USA; 6 Internal Medicine, Shadan Institute of Medical Sciences, Hyderabad, IND

**Keywords:** palliative care, end of life care, hospice care, home care, cbc wonder database, mortality trends

## Abstract

Introduction

This study aims to examine the disparities in the place of death for patients due to thyroid neoplasms and understand the mortality trends. The study also aims to assess the influence of factors like age, gender, geography, and race, thus allowing for the assessment and improvement of end-of-life and palliative care.

Methodology

The study analyzes thyroid cancer mortality trends from 1999 to 2020 using the Centers for Disease Control and Prevention Wide-ranging Online Data for Epidemiologic Research (CDC WONDER) database, taking into consideration locations of death, medical facilities, home and hospice care, and others. Additional categories such as race, gender, and U.S. census regions were variables chosen to segregate the deaths. Microsoft Excel (Microsoft Corporation, Redmond, Washington, United States) and autoregressive integrated moving average (ARIMA) modeling were used for data analysis.

Results

The study revealed that around 50% of thyroid cancer patients in the United States passed away at home or in hospice settings, while the other 50% died in medical facilities or nursing homes. Patients aged 65-74 and 75-84 were more likely to die at home or in hospice, and males had a higher likelihood of dying in these settings compared to females. Geographically, individuals in the South and West regions were more inclined to die at home or in hospice. Additionally, racial disparities were observed, with Black or African Americans being less likely than Whites to die in home or hospice settings.

Conclusions

Socio-demographic factors play a major role in shaping end-of-life care, underscoring the need for tailored interventions. There is also a need for more refined early diagnostic techniques for smaller, localized tumors. Future studies should investigate the relationship between profession and income and the incidence and mortality of thyroid cancer.

## Introduction

A death where the patient passes away in their preferred setting, with the least amount of pain possible, and with their wishes honored, would be the most appropriate kind. Maximizing patients' quality of life, reducing their suffering, and assisting them in making decisions in line with their values and preferences, such as life extension, quality of life, place of care, and place of death, are the main goals of patient-centered end-of-life care [1].

Palliative care is defined as the treatment of symptoms and pain for incurable illnesses; as such, it can be given to individuals with any type of illness, not just those who are nearing the end of their lives. On the other hand, modern end-of-life care encompasses what is recognized as a more comprehensive approach to patient care, which includes social, emotional, and frequently religious support in addition to palliative care [2]. Each year, palliative care is needed for 20 million people worldwide as they near death. The World Health Organization recognizes palliative care and the alleviation of patients' and families' needless suffering as an urgent humanitarian need. Such care is a crucial component of a society's wider healthcare system [3].

It is a general belief for many that dying at home or in a hospice is better than passing away in a hospital or skilled nursing facility. However, while most of the symptoms at the end of life can be managed at home or in a hospice facility with the right assistance, these symptoms can be managed more effectively in a hospital or skilled nursing facility [4].

Malignant neoplasm of the thyroid is a prevalent endocrine malignancy. Statistically, papillary thyroid carcinoma (PTC), which has an excellent prognosis, accounts for 80%-85% of all thyroid malignancies. Follicular thyroid cancer (FTC) is a contributing factor in 10% to 15% of cases. PTC and FTC are considered relatively low-risk cases because of their good rate of survival (>98%) and low rate of recurrence (<5%). Anaplastic thyroid carcinoma (ATC; 1%-2%) is the least common histotype, which progresses quickly and has a grim prognosis, with a mean survival period of two to six months. Lastly, medullary thyroid carcinoma, which makes up approximately 4% of thyroid cancers, develops from thyroid parafollicular calcitonin-producing cells [5].

Thus, finding disparities in the place of death for patients with malignant neoplasms of the thyroid may present a chance to improve patient care during the final stages of life.

Aims and objectives

To evaluate the disparities in thyroid neoplasm deaths at home or hospice, and medical facilities or nursing homes in the USA, with a focus on how these disparities vary based on factors such as age, gender, census region, and race, using data from 1999 to 2020 from the Centers for Disease Control and Prevention Wide-ranging Online Data for Epidemiologic Research (CDC WONDER) database, to assess how these variations have changed over time, to identify any patterns or trends that may emerge, and to provide insights that can help improve end-of-life care.

## Materials and methods

We conducted a retrospective study to investigate disparities in the place of death in malignant neoplasms of the thyroid. Data for this study was obtained on October 13, 2023, in a single day from the CDC WONDER database, which provides comprehensive mortality statistics in the United States.

Database description

The CDC WONDER is a simple, menu-driven system that makes the Centers for Disease Control and Prevention's (CDC) information resources available to public health professionals and the general public, as well as providing users with access to a wide range of public health information.

The CDC WONDER assists us with extracting data based on each category. It served as the primary source for this study, providing comprehensive and reliable mortality data.

Data collection

We focused on the underlying causes of death, utilizing the data spanning from 1999 to 2020. The dataset was organized based on the Underlying Cause of Death by Bridged-Race Categories, using the relevant International Classification of Diseases, 11th Revision (ICD-11) code(s), as indicated within our research group (ICD-11 code(s) provided by the group). The ICD-11 code(s) and their full form were integral to the selection process [6].

Based on our topic, we selected locations of death, medical facilities, home and hospice care, and others. We also chose additional categories such as race, gender, and U.S. census regions to separate deaths caused by malignant tumors of the thyroid gland to obtain statistical data on the highest and minimum number of deaths in the aforementioned categories [7]. For our chosen disease, the ailment is designated as C73 according to ICD-11.

Data segmentation

The study encompassed demographic parameters of individuals of all age groups, genders, races, and those residing in different U.S. census regions [8].

Inclusion criteria

The time frame of the study includes people whose causes of death between the years 1999 and 2020 were listed as malignant thyroid neoplasms. It also emphasizes cases that have been specifically coded using the ICD-11 criteria for thyroid cancer [9].

Exclusion criteria

The study excluded non-relevant cases that did not fit one or more of the ICD-11 codes for malignant neoplasm of the thyroid. As assessed by the data quality guidelines of the CDC WONDER database, instances with insufficient or unreliable data were also disregarded.

Data analysis

Following data extraction from CDC WONDER, the relevant information was exported into a Microsoft Excel sheet for further analysis. We summarized the total number of deaths for all years under consideration, with a specific focus on the predefined groups mentioned within our research.

Statistical analysis was conducted using the autoregressive integrated moving average model (ARIMA) [10]. The results of this analysis provided insights into the mortality trends associated with malignant neoplasm of the thyroid within the specified demographic groups.

## Results

An aggregate of 36,605 deaths due to thyroid neoplasms was obtained from the CDC WONDER database. Around 17,328 patient deaths happened at home or under hospice care; 17,539 deaths within a medical facility or nursing home; and 1,918 deaths were due to other causes. The number of deaths was further subcategorized based on age, gender, census region, and race.

Table 1 shows the total number of deaths due to malignant thyroid neoplasms at home or under hospice care, in a medical facility or nursing home, or due to other causes. The majority of deaths occurred in the 75-84 age range, which was 5,150 deaths at home or hospice. Additionally, the age range of 15 to 24 years had 20 deaths at home or hospice, which may be related to the lower incidence of thyroid neoplasms in the younger population. Similarly, women died at a higher rate than men did; 9,894 deaths in hospitals or nursing homes were the highest for women, while 751 deaths from other causes were the lowest for men. The maximum number of deaths (6,284) was seen in the Southern census region. 

**Table 1:** The total number of deaths due to malignant thyroid neoplasms at home or under hospice care, medical facility, or nursing home, or due to other causes

Demographic variables	Home or hospice (n =17328)	Medical facility or nursing (n=17359)	Others (n=1918)
Ten-year age groups			
15-24 years	20	27	0
25-34 years	92	134	12
35-44 years	332	413	33
45-54 years	1206	1398	128
55-64 years	2959	3031	301
65-74 years	4575	4279	445
75-84 years	5150	4846	564
85+ years	2988	3181	410
Gender			
Female	9478	9894	1167
Male	7850	7465	751
Census Region			
Census Region 1: Northeast	2833	4145	221
Census Region 2: Midwest	3804	4000	400
Census Region 3: South	6284	5333	814
Census Region 4: West	4407	3873	462
Race			
American Indian or Alaska Native	68	90	0
Asian or Pacific Islander	759	996	59
Black or African American	1275	1958	156
White	15217	14312	1696

Table 2 shows the predictors of deaths at home or hospice due to malignant thyroid neoplasms. Using univariate logistic regression, the likelihood of deaths in the subcategories was determined. Using the 85+ age range as the reference, those in the 65-74 age range were 1.164 times more likely to die from malignant thyroid neoplasms. Patients in the 25-34 age range were 0.757 times less likely to die from malignant thyroid neoplasms compared to the reference. Similarly, males were 1.115 times more likely to die than females. Taking the Northeast region as a reference, patients from the Midwest, South, and West were more likely to die from malignant thyroid neoplasms, with the maximum likelihood of deaths being 1.575 more likely in the Southern census region. Additionally, taking Asians or Pacific Islanders as the reference range, patients of White origin were 1.321 times more likely and African Americans were 0.838 times less likely to die from malignant thyroid neoplasms.

**Table 2:** The predictors of deaths at home or hospice due to malignant thyroid neoplasms The significant P-values (<0.05) are marked with an asterisk (*)

Variables	Univariate logistic regression		
	Odds ratio	95% Confidence interval	P-value
Age			
15-24 years	0.89	(0.498, 1.59)	0.694
25-34 years	0.757	(0.581, 0.987)	0.04*
35-44 years	0.895	(0.77, 1.04)	0.146
45-54 years	0.95	(0.868, 1.039)	0.261
55-64 years	1.067	(0.996, 1.144)	0.066
65-74 years	1.164	(1.092, 1.24)	<0.001*
75-84 years	1.144	(1.076, 1.217)	<0.001*
85+ years	1.0 (Reference)		
Gender			
Male	1.115	(1.07, 1.162)	<0.001*
Female	1.0 (Reference)		
Census Region			
Census Region 1: Northeast	1.000 (Reference)		
Census Region 2: Midwest	1.332	(1.25, 1.421)	<0.001*
Census Region 3: South	1.575	(1.485, 1.671)	<0.001*
Census Region 4: West	1.567	(1.471, 1.669)	<0.001*
Race			
American Indian or Alaska Native	1.05	(0.756, 1.459)	0.77
Black or African American	0.838	(0.746, 0.942)	0.003*
White	1.321	(1.2, 1.454)	<0.001*
Asian or Pacific Islander	1.000 (Reference)		

**Figure 1:**
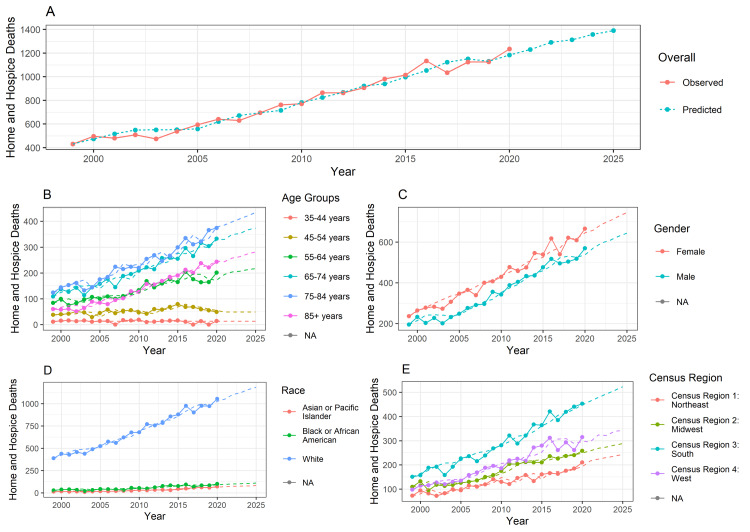
The trends of deaths at home or hospice due to malignant thyroid neoplasms from 1999 to 2020

Figure 1 showcases the trends of deaths at home or hospice due to malignant thyroid neoplasms from 1999 to 2020. Line charts showing overall deaths as well as deaths by age, gender, race, and census region were generated using the ARIMA model. With the available training data from the years 1999 to 2020, forecasting was done for up to 2025, and thus the prediction for the next five years was also made.

As seen in Figure 1A, the overall trend for deaths due to malignant thyroid neoplasms at home or hospice has gradually increased from 1999 to 2020. By the year 2025, the overall number of deaths will likely reach around 2250, based on the predictions. In Figure 1B, there has been a gradually increasing trend in deaths in the age groups 75-84 and 65-74 while deaths in the age groups 35-44 and 45-54 remain stable. Figure 1C showcases gradually rising trends in deaths for both genders, with a greater incidence of deaths among females. Figure 1D demonstrates a steadily rising trend in deaths in the White population in contrast to the constant pattern seen in other races. Figure 1E shows that the Southern census region has the highest number of deaths, which is progressively rising in comparison to the other census regions.

## Discussion

To study disparities in the place of death of malignant neoplasms of the thyroid gland, 22-year data was collected from CDC WONDER. There were 17,328 home or hospice deaths, 17,359 in medical or nursing facilities, and 1,918 other deaths. We highlight the important problem of racial differences in thyroid cancer outcomes in our discussion.

In terms of race, American Indians and Alaska Natives died in smaller numbers (n=68), while White people died at home or in hospice care the most (n=15,217). The American Indian or Alaska Native population reported the fewest deaths (n=90), while the White population reported the greatest number of deaths (n=14,312) in the hospital or nursing home. In other situations, American Indians or Alaska Natives reported the fewest deaths (n=0), while White people reported the most deaths (n=462). Although thyroidectomy is still the most common treatment for metastatic thyroid cancer, a significant fraction of patients still undergo radiotherapy alone, even despite national guidelines that recommend surgery. Our study thus highlights the larger context of racial differences in thyroid cancer outcomes by integrating these findings with data on mortality patterns in malignant neoplasms of the thyroid gland.

Data from Lim et al. also show greater death rates for White people and significantly lower rates for American Indians and Alaska Natives, further supporting our evidence in showing how stark the discrepancies are [11]. Similarly, the results of the medullary thyroid cancer (MTC) study highlight how important the Black race is for treatment outcomes as well as overall survival. Megwalu et al. further found that Black race is a poor prognostic predictor for thyroid cancer that is unrelated to advanced disease stage [12]. However, Angelina Magreni et al. discovered a steady rise across all racial and ethnic categories. The increase was significantly higher in non-Hispanics than in Hispanics, even though they found no discernible difference between the White and Black populations [13].

According to our research, more women died at home or in hospice care (n=9,478), in medical or nursing facilities (n=9,894), or in other places (n=1,167). This is comparable to the research by Reza Rahbari et al., which found that women who are in the reproductive age group have a threefold increased risk of thyroid cancer [14].

The age groups with the highest death rates were those for home or hospice care, medical or nursing care, and others, all falling between the ages of 75 and 84. However, the research conducted by IV Shevchyk et al. showed the likelihood of receiving less than a total thyroidectomy increased with age, with patients aged 85 and above being three times more likely to receive less than a total thyroidectomy than younger patients [15].

The greatest number of deaths in home or hospice settings was reported by Census Region 3: South (n=6,284), while the lowest number was reported by Census Region 1: Northeast (n=2,833). Census Regions 3 and 4 show that the South and West, respectively, had the greatest and lowest numbers of deaths in hospitals (n=5,333) and nursing homes (n=3,873). In the ‘Others’ category, Census Region 3: South had the most deaths (n=814), while Census Region 1: Northeast had the fewest (n=221). Numerous factors, such as population demographics, healthcare infrastructure, and cultural or regional preferences regarding end-of-life care, could be responsible for these regional disparities. On the contrary, the incidence rates of thyroid cancer in the U.S. were examined in a study by Robert Udelsman and Yawei Zhang and found that from 1999 to 2009, the Northeast had the highest rates, while the South had the lowest [16].

Udelsman and Zhang also found the incidence of thyroid cancer to be strongly correlated with the densities of general surgeons, endocrinologists, and cervical ultrasonography. Furthermore, they also found that a small portion of this variability in incidence rates was further explained when taking median household income into account, raising the possibility that healthcare resources and thyroid cancer incidence are related, especially in the Northeastern states [16]. Hence, future studies should also consider the profession of the patients and median household income as areas of interest.

Our study found a steady increase in incidence rates from 1999 to 2020, with a small dip after 2015. This can be contrasted with the study by Megwalu et al., which found increasing incidence rates of localized and smaller thyroid tumors until 2014, followed by a decline, contrasted by their finding of inclining incidence-based mortality rates, indicating that although management practices may be changing, fewer small, indolent tumors are being diagnosed rather than more advanced cases [17]. This may indicate that, despite changing management practices, smaller tumors are not being diagnosed as well as the more advanced cases.

In our study, a higher number of females died in homes or hospices, medical or nursing facilities, or others. This is in contrast to the study by Wirth et al., who studied the trends in Zurich from 1980 to 2016 and found no appreciable differences in thyroid cancer incidence rates between men and women but a notable rise in cases, mainly due to the papillary subtype [18]. On the other hand, mortality rates fell for both genders. The study acknowledges the possibility of a true increase in thyroid cancer cases but also raises the possibility of more indolent thyroid cancers being diagnosed and further emphasizes the need for targeted diagnostic strategies to prevent overdiagnosis [18].

Limitations

In evaluating this study, it is essential to highlight a few limitations. The research exclusively used the CDC WONDER database, an aggregate-level data source, which might not capture all the nuances, especially in cases of underreporting or misclassification. Although the 22-year timeframe (1999 to 2020) offers an extensive overview, it doesn't account for developments post-2020. The focus on the U.S. may not represent global disparities and might overlook regional variations within the country. While the study incorporated demographic factors such as age, race, and gender, deeper subtleties within these groups might be absent. Other potentially influential factors, like healthcare policies, socio-economic variables, patient preferences, and access to care, may need to be thoroughly represented. Additionally, the observational nature of the research precludes establishing definitive causality between observed disparities and potential influencers. Future studies could benefit from incorporating multiple databases for a more comprehensive perspective, expanding the geographical scope to understand global disparities better, and delving deeper into individual patient data to uncover overlooked nuances.

## Conclusions

Over 22 years (1999 to 2020) as revealed by the CDC WONDER database, significant disparities were observed regarding the place of death for patients with malignant neoplasms of the thyroid gland in the U.S. Approximately 50% of the recorded deaths took place at home or in hospice, equating to the 50% observed in medical facilities or nursing homes. Age emerged as a crucial determinant, with patients aged 65-74 and 75-84 showing odds ratios of 1.164 and 1.144, respectively, in contrast to those aged 85+. Gender also influenced outcomes, males, bearing an odds ratio of 1.115, were 11.5% more likely to die at home or in hospice settings than females. Regionally, patients in the South and West demonstrated a higher inclination towards home or hospice deaths. Additionally, racial data indicated that Black or African Americans were 16.2% less likely than Whites to die in these settings. These insights emphasize the importance of socio-demographic factors in shaping end-of-life care, underscoring the need for tailored interventions. There is also a need for more refined early diagnostic techniques for smaller, localized tumors. Future studies should investigate the relationship between profession and income and the incidence and mortality of thyroid cancer.
